# Analyses of the Updated “Animal rDNA Loci Database” with an Emphasis on Its New Features

**DOI:** 10.3390/ijms222111403

**Published:** 2021-10-22

**Authors:** Jana Sochorová, Francisco Gálvez, Roman Matyášek, Sònia Garcia, Aleš Kovařík

**Affiliations:** 1 Institute of Biophysics, Academy of Sciences of the Czech Republic, 61265 Brno, Czech Republic; sochorova@ibp.cz (J.S.); matyasek@ibp.cz (R.M.); 2 Bioscripts—Centro de Investigación y Desarrollo de Recursos Científicos, 41012 Sevilla, Spain; franciscogp@bioscriptsdb.com; 3 Institut Botànic de Barcelona (IBB-CSIC), Passeig del Migdia s/n, 08038 Barcelona, Spain; soniagarcia@ibb.csic.es

**Keywords:** ribosomal DNA, rDNA, rRNA genes, nucleolar organizer regions, karyotype, sex chromosome, B chromosome, Ag-NOR, database, animals

## Abstract

We report on a major update to the animal rDNA loci database, which now contains cytogenetic information for 45S and 5S rDNA loci in more than 2600 and 1000 species, respectively. The data analyses show the following: (i) A high variability in 5S and 45S loci numbers, with both showing 50-fold or higher variability. However, karyotypes with an extremely high number of loci were rare, and medians generally converged to two 5S sites and two 45S rDNA sites per diploid genome. No relationship was observed between the number of 5S and 45S loci. (ii) The position of 45S rDNA on sex chromosomes was relatively frequent in some groups, particularly in arthropods (14% of karyotypes). Furthermore, 45S rDNA was almost exclusively located in microchromosomes when these were present (in birds and reptiles). (iii) The proportion of active NORs (positively stained with silver staining methods) progressively decreased with an increasing number of 45S rDNA loci, and karyotypes with more than 12 loci showed, on average, less than 40% of active loci. In conclusion, the updated version of the database provides some new insights into the organization of rRNA genes in chromosomes. We expect that its updated content will be useful for taxonomists, comparative cytogeneticists, and evolutionary biologists.

## 1. Introduction

Ribosomal DNA (rDNA) loci encoding 5S and 45S (18S-5.8S-28S) rRNA genes are vital components of eukaryotic chromosomes [1]. Ribosomal DNA also plays a pivotal role in nuclear organization by assembling the nucleolus [2] and epigenetic stabilization of the genome [3]. Ribosomal DNA loci, together with centromeres and telomeres, are distinguished features in eukaryotic chromosomes. While telomeres and centromeres tend to occupy specific chromosome regions, rDNA loci are often found at variable positions and numbers. These features make rDNAs important karyotypic and phylogenetic markers. Although the relationship between nucleolar organizer regions (NOR) and distinct chromosome segments had already been proposed by Barbara McClintock [4], the composition of NORs and rDNA loci in general were only deciphered with the aid of in situ hybridization experiments in the 1970s [5,6,7]. However, the invention of fluorescence labeling of probes [8] triggered enormous progress in the molecular cytogenetics of rDNA loci. Currently, many protocols are available for fluorescence in situ hybridization (FISH) of rDNA in different systems [9,10,11]. Ribosomal DNA may occur at any place on chromosomes, whereas some preference exists for its subtelomeric position, at least in the case of 45S rDNA; however, 5S rDNA is more variable [12,13,14]. Aside from its standard position on autosomes, there are also examples of rDNA occurrences on sex chromosomes [15,16] and B chromosomes [17,18]. In addition, rDNAs may even be largely pseudogenized in some large genomes [19,20]. 

For many years, cytogenetic data on rDNA loci remained scattered across the literature or compiled into lists available only in hard copy. This motivated authors to generate public databases summarizing information about the number and position of rDNA loci in plants [21] and animals [13]. Here, we report on the first update of the animal rDNA loci database (http://www.animalrdnadatabase.com), which went live in August 2021 and which collates data from 1040 original publications. In the analyses, we addressed questions on: (i) the diversity of rDNA loci numbers, (ii) the occurrence of rDNA loci in chromosomes with limited or no recombination capacity, and (iii) the relationship between 45S rDNA loci numbers and their activity.

## 2. Methods

### 2.1. Data Collection

The database includes data collected from the literature until March 2021 (1040 papers). Papers were retrieved from the bibliographical databases Thomson Reuters WOS, MEDLINE/PubMed and Google Scholar. Queries used for search were: “rDNA”, “rRNA”, “chromosome”, “in situ hybridization”, “FISH”, “hybridization”, and all combinations thereof. We also searched in the references of each of the retrieved papers. The data come mostly from fluorescent in situ hybridization (FISH) experiments, although a small number of older entries were obtained from radioactive hybridization methods. The assessments of the number and position of rDNA signals were taken from the authors’ evaluations when explicitly mentioned and manually counted when only shown in chromosome pictures.

New features for an advanced search were added, such as information about the type of chromosome-bearing rDNA locus (autosome, sex chromosome, and B chromosome) and information about the number of Ag-NOR signals (reporting active NORs detected by silver nitrate staining). Papers containing unclear information about the number of Ag-NOR signals or citing another research paper analyzing these data were disregarded. In the text, the terms “locus”, “site”, and “signal” are used interchangeably.

### 2.2. Website Construction

The tabular database structure comprising the rDNA and literature data was created in SQL (Structured Query Language) tables on a MySQL server. Each table had its own different field type and size. The initial spreadsheet table in which the data were compiled was exported to a CSV (comma-separated values) file. A unique ID was given to each entry, together with the date and time of the export and the version of the database. Then, it was imported (current version is the seventh import) into the SQL database (www.animalrdnadatabase.com). The website was programmed in HTML (HyperText Markup Language), CSS (Cascading Style Sheets), and JS (JavaScript) to visualize the website. The custom functions in PHP (PHP: Hypertext Preprocessor) were written to query the database and process and display the data. 

### 2.3. Data Analyses

We carried out basic statistical analyses of the results (locus number average, median, range for all groups) using MS Excel and RStudio [22]. The Spearman’s correlation coefficient (Rs) was counted using “RANK(AVG) and “CORREL” commands; values above 0.04 (in absolute values) were considered significant (*p* < 0.05). The statistical support for differences between the groups was calculated using the ANOVA (MS Excel function) and Mann–Whitney (online program: Mann_Whitney_U_test_calculator [23]) tests. The Levene’s equality test (online program: [24]) was applied to determine the homogeneity of variances in locus numbers between the groups. We also analyzed rDNA locations on different types of chromosomes (autosomes, sex chromosomes, and B chromosomes) in the different groups of animal species. In box plot graphs (constructed in RStudio), values < Q1 − 1.5 × IQR and values > Q3 + 1.5 × IQR are outliers (IQR computed as the interquartile range of the input data).

## 3. Results and Discussion

### 3.1. Database Content

**Figure 1:**
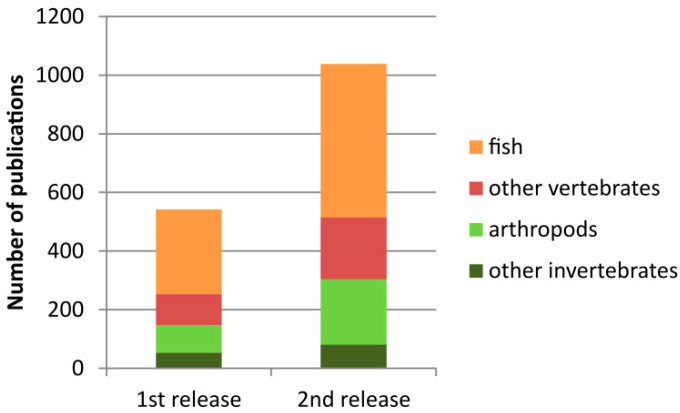
Increased amount of literature in the current version of the animal rDNA loci database.

Since its first release in 2016, the number of entries in the current version has almost doubled (Figure 1). The cytogenetic information about rDNA (either 45S or 5S) is currently available for more than 2500 species (Table 1). The largest groups include arthropods (1068 karyotypes, 38%) and fish (953 karyotypes, 34%). 

The remainder, including mammals, amphibians, birds, mollusks, and reptiles, among others, represents 28% of the database content (796 karyotypes). Although birds are the second largest class of vertebrates (after fish), they were severely underrepresented in a previous release. The current release has improved the situation in that it contains data for 83 bird species, mainly collected from a recent publication of Degrandi et al. [26]. Arthropods seem to be well represented in the database, but considering that they are the largest animal group (>1.3 million species, about 95% of all animals, source: [25,27]), their representation is relatively low (<0.1% species). It is, therefore, not surprising that the data for arthropods are steadily expanding. For example, Stahlavsky et al. [28] recently published FISH data on 18S rDNA loci for 74 scorpion species, which were not represented in the previous release. The best represented group in terms of diversity is fish, which also has relatively balanced entries for both 45S and 5S loci (Table S1). Considering animal diversity (Table 1), our information about rDNA loci is still fragmentary, but a continuous rise in publications can be envisaged. 

### 3.2. The Number of rDNA Loci Is Variable, but Most Species Tend to Maintain a Low Locus Number 

**Figure 2:**
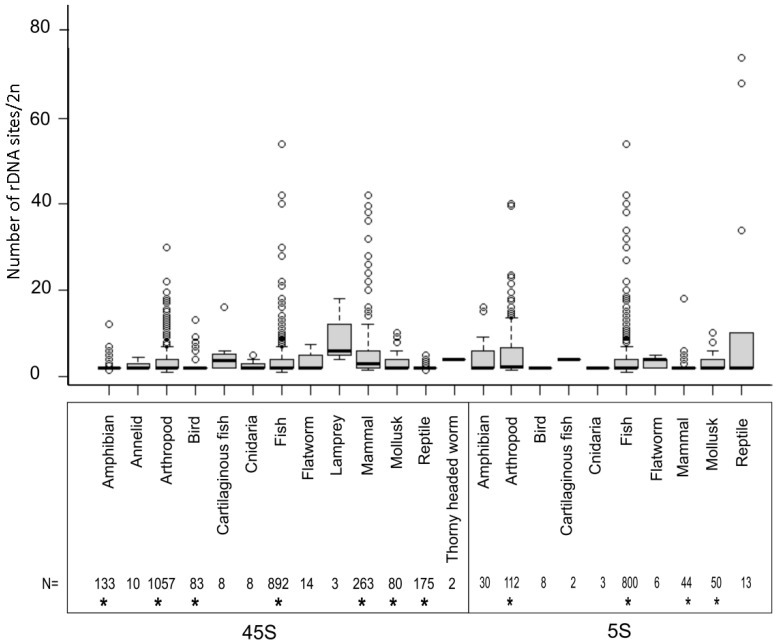
Number of 45S and 5S rDNA sites per diploid cell in different animal groups. The sample size is shown below the diagram. Boxes represent Q1, median, Q3. Outliers marked by circles were calculated from the interquartile range (IQR): < Q1−1.5 × IQR and > Q3 + 1.5 × IQR. Asterisks (*) below the numbers mark groups in which statistical tests were carried out (Table S1).

The number of rDNA loci is an important characteristic of every karyotype. This can be relatively easily determined by counting the probe hybridization signals in metaphase chromosomes, although this depends greatly on the chromosome size and the quality of materials for the analysis. Figure 2 shows the distribution of locus numbers in individual groups. In all major groups that could be statistically evaluated (those represented by at least 40 karyotypes), the medians ranged from two to three sites (both 5S and 45S) per diploid karyotype (Table S1). 

It is, therefore, not surprising that in many genera karyotypes with two NORs (per diploid genome), this has been considered the ancestral condition [29,30]. Mammals had a slightly larger number (5.51 average; 3.0 median) of 45S loci compared to other animals (*p* < 0.05, Mann–Whitney U test, Table S2). The largest number of 5S loci was observed in arthropods (6.46 average; 2.25 median; *p* < 0.05, Mann–Whitney U test). However, in each major group, prominent outliers were observed, with a locus number significantly deviating from the average. The number of 45S loci (per 2C) ranged 1–54 in fish (892 karyotypes), 1–42 in mammals (263), 1–30 in arthropods (1057), 2–13 in birds (83), 1–12 in amphibians (133), 2–9 in mollusks, and 1–7 in reptiles (175) among the most (>50 species) represented groups (Table S1). The 5S loci ranged 1–54 in fish (800 karyotypes), 1–40 in arthropods (112), 2–18 in mammals (44), and 2–10 in mollusks (50). About 2.5% of karyotypes harbored a single rDNA site per diploid set, mostly occurring in heterogametic species bearing a single locus on sex chromosomes and none on the autosomes (specifically, 0.46% for 5S and 2.01% for 45S loci). Two large groups (arthropods and fish) showed near equal variability in the number of 45S rDNA sites (Levene’s test, *p* > 0.05 (Table S3)), suggesting that differences between groups tend to diminish as the sample size increases and that a group’s diversity is likely the main factor influencing locus number variability. Remarkably, a several-fold variation in locus number was observed at the genus level in groups as phylogenetically divergent as grasshoppers [31] or mice [32]. Although the time scales of these shifts are unknown, they demonstrate the overall well-known dynamics of rDNA in chromosomes [13,33,34]. 

**Figure 3:**
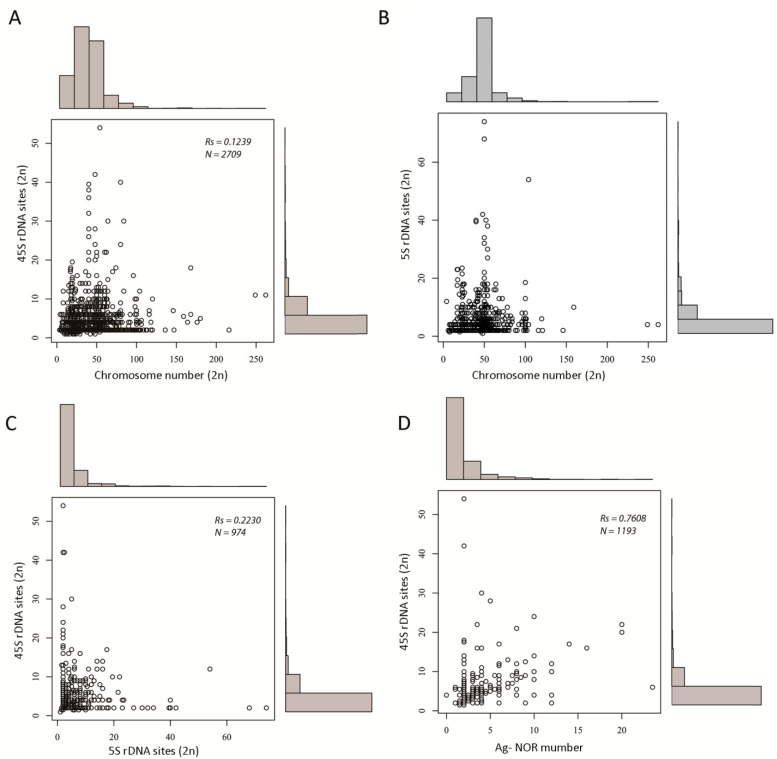
Scatter graphs showing relationships between rDNA locus number and other variables including chromosome number and number of Ag-NOR signals. (**A**) A plot of 45S rDNA and chromosome number. (**B**) The same as in (**A**) but for 5S rDNA. (**C**) A plot of 5S and 45S rDNA numbers. (**D**) A plot of the number of Ag-NOR signals and number of 45S rDNA loci. Legend: Rs, Spearman’s correlation coefficient; N, number of data points. Details of statistical tests are in Table S2.

There was weak to no correlation between the number of rDNA loci and chromosome number (Figure 3A,B and statistical tests (Table S4)). This apparently holds true even for species with an extremely large (typically >160/2C) number of chromosomes, such as lampreys [35,36,37], some arthropods (e.g., the *Austropotamobius* genus members [38]), and some fish (e.g., *Acipenser* [39]), which tend to keep their rDNA locus (45S) numbers close to the median (low). 

There was a weak correlation between the number of 5S and 45S loci (Spearman’s Rs = 0.22, N = 974 (Figure 3C and Table S4)), which is in line with our previous study using about half of the amount of data [13]. Additionally, both parameters were not correlated at the group level, for instance in arthropods (Rs = 0.03) and fish (Rs < 0.01). Strikingly, a similarly weak correlation (Spearman’s Rs = 0.24) was observed between 18S and 5S copy numbers in human populations [40], indicating independent evolution of these loci, in general. This, however, does not exclude common shifts at the population level [41]. 

### 3.3. The Occurrence of rDNA Loci on Sex and B Chromosomes

The new database option allows the retrieval of information about the presence/absence of sex chromosomes and B chromosomes in the karyotype. About 45% of karyotypes in the database contain sex chromosomes, and 4% of the karyotypes harbor a variable number of B chromosomes. Considering the whole database, the rDNAs (45S and 5S) are located on autosomes (88%), sex chromosomes (11%), and B chromosomes (1%). All mammals and birds carry sex chromosomes, whereas 2.5% of mollusks, 10.9% of fish, and 68.6% of arthropod karyotypes carry sex chromosomes.

Table 2 shows the frequency of sex chromosomes in individual groups and the occurrence of rDNA in them. Nearly all types of sex chromosomes may carry rDNA loci (6.9% X, 2.5% Y, 1% W, and 0.6% Z). Among the heterogametic karyotypes, almost 33% of fish, 24% of arthropods, and 6% of mammals carried rDNA (5S or 45S) on sex chromosomes, suggesting a lower frequency of sex chromosome-linked rDNA in mammals compared to the other two groups (chi-square test, *p* < 0.001, Table S5).

In addition, many other insect species only bear rDNA on sex chromosomes (option “sex chrom. number” on http://www.animalrdnadatabase.com/), and there are also examples of 45S rDNA on both X and Y chromosomes in a single karyotype [42,43]. These observations support the hypothesis of the possible ancestral character of sex-linked NORs in insects as proposed in previous studies [15,29,44,45,46,47]. It is possible that there is selection towards a preferential position of NOR on sex chromosomes in insects. In support of this, the functionality of sex chromosome-linked NORs has been demonstrated by cytogenetic [48,49,50] and molecular methods [51]. NORs were also proposed to be involved in sex chromosome pairing and disjunction [30], and rDNA intergenic spacers might even participate in this process [52]. 

Next, we analyzed the mutual position of 5S and 45S rDNA on sex chromosomes. We selected karyotypes for which the positions of both loci are known (805 karyotypes) and where at least one of the loci (either 5S or 45S) occurs on a sex chromosome (37 karyotypes). There are three possibilities: (i) 5S rDNA is on a sex chromosome, and 45S rDNA is on an autosome; (ii) 5S rDNA is on an autosome, and 45S rDNA is on a sex chromosome; and (iii) both 5S and 45S rDNA are localized on sex chromosomes. The 5S and 45S loci may occur on the same [53] or different [54] sex chromosomes. However, the possibility of both 5S and 45S rDNA in the same sex chromosome could not be evaluated due to the reduced species dataset with this feature. It is evident that karyotypes with both 5S and 45S on sex chromosomes were relatively frequent (Figure S1). Colocalization of 5S and 45S was also observed on an M chromosome in the insect *Hypselonotus fulvus* [55]. M chromosomes are believed to represent a transition state from autosome to sex chromosome, suggesting an autosomal origin of sex-linked chromosomes. Certainly, rDNA mobility could also be driven by transposons, which may spread rDNA from autosomes to sex chromosomes and vice versa [56,57].

The frequency of rDNA on B chromosomes is shown in Table 3. Although the numbers are low due to the relatively low representation of species with B chromosomes in the database, it is clear that the rDNA location of at least one locus (5S or 45S) in B chromosomes is quite frequent in many of the amphibians, arthropods, fish, and mammals analyzed. Of note, in grasshoppers, rDNA on B chromosomes seems to be transcribed and form active nucleoli [58], so despite B chromosomes being seen as sometimes unnecessary or even harmful, it is also possible that they include important regulatory regions of the genome which might be under positive selection.

In sum, the fact that rDNA on sex and B chromosomes is not an uncommon configuration raises a number of questions about the homogeneity of these particular chromosomes, which is supposed to be reduced compared to autosomes due to reduced meiotic recombination. 

### 3.4. Peculiarity of Bird and Reptile Karyotypes: Frequent Occurrence of rDNA on Microchromosomes 

The animal rDNA loci database also provides information on whether rDNAs occur in the distal, proximal, or interstitial position or on microchromosomes. These are very small chromosomes (usually less than 20 Mb in size and less than 2.5 µm long) found in all bird karyotypes, most reptiles, some fish, and rarely in other animals [59]. In our data sets, 100% of bird, 71% of reptile, and 4% of amphibian karyotypes contain microchromosomes (Table 4). 

The localization of at least one 45S rDNA site on microchromosomes was observed in 85.5% of birds (out of 83 karyotypes with microchromosomes), 27.8% of reptiles (out of 125 karyotypes with microchromosomes), and 66.7% of amphibians (but only out of six karyotypes with microchromosomes). In contrast to other groups, most bird karyotypes did not display large variability in rDNA locus numbers, and 45S rDNA was typically located on a single pair of microchromosomes [60,61], although significant exceptions exist even in this group. For example, peregrine falcons (*Falco peregrinus*) harbor 16 45S rDNA sites per diploid cell, all located on microchromosomes [62]. Interestingly, microchromosomes (11 pairs) are less abundant than the macrochromosomes (14 pairs) in the *F. peregrinus* karyotype (2n = 50), underlining a relatively strong trend towards the preferential location of 45S loci on microchromosomes. The 45S rDNA is only exceptionally found on macrochromosomes, such as among birds of prey who belong to the subfamily Buteoninae [63]. This configuration is, however, believed to be a derived condition arising from chromosome rearrangements [26]. Birds are also distinct in having little or no karyotypic variability (most diploid counts range 74–86 [64]), resonating with the stasis of the rDNA loci in this group. 

In contrast to birds, only 27.8% of reptile karyotypes show 45S rDNA loci on microchromosomes. Crocodylidae are considered the evolutionary closest extant reptile family to birds, with both lineages separating c. 250 million years ago [65]. However, in Crocodylidae, 45S rDNA is located on small chromosomes [66,67] that are not considered microchromosomes (Deakin and Ezaz 2019). However, more divergent groups less close to birds than crocodiles (such as snakes, lizards, and turtles) do present microchromosomes that carry rDNAs, albeit at a lower frequency than birds. Additionally, a considerable variation in NOR positions on macro- and microchromosomes was encountered at the genus level [68]. This suggests that rDNA localization in microchromosomes arose several times in the evolution of the reptile lineage. It is possible that there is a selection for the preferential position of 45S rDNA in microchromosomes. These are usually highly GC rich [69], gene rich [70], and recombinationally active [71]. It is likely that 45S rDNA tends to amplify in chromosome niches with increased recombinogenic activity such as in the (sub)telomeric regions [12,14] and microchromosomes. 

As for 5S rDNA in microchromosomes, the information is much more limited as compared to 45S rDNA, although it has been identified in several birds [72,73], reptiles [66,74], and amphibians [75]. 

### 3.5. Relationship between 45S rDNA Locus Number and NOR Activity

The rDNA expression is under epigenetic control, and only a subset of genes (and loci) are active in cells at a given time interval [76]. At the cytogenetic level, the activity of NORs has been studied by silver (Ag-NOR) staining in various systems and cell types [48,77,78,79]. The number of Ag-NOR signals is an important marker of cell proliferation in cancer biology [80]. Despite advances in understanding the molecular mechanisms of rDNA expression, cytogenetic information about the NOR activity is still valuable since the silencing of rDNA is a chromosome-specific epigenetic phenomenon [81]. The new database option “Ag-NOR” returns information about the number of active NORs in karyotypes. This is based on the silver nitrate staining of the nucleolar organization region (NOR)-associated proteins, producing a dark region wherein the silver is deposited, and denoting the activity of rRNA genes within the NOR [82]. The database contains information about the number of Ag-NOR signals in 1192 karyotypes, which is almost half of its total content. Most data come from fish (606 karyotypes) and arthropods (300); the rest comprise diverse groups including mammals, birds, and reptiles. 

**Figure 4:**
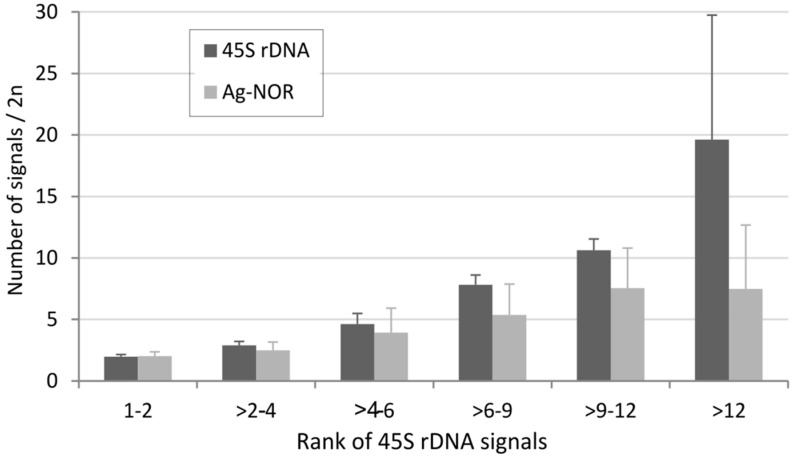
Relationship between the number of 45S rDNA loci and the number of Ag-NOR signals. The average number of Ag-NOR signals and loci was counted in each group differing in locus number (x-axis) and expressed as an average (y-axis). The number of karyotypes in each group was as follows: 753 (group “1–2”), 60 (“>2–4”), 237 (“>4–6”), 44 (“6–9”), 32 (“>9–12”), and 31 (“>12”).

We analyzed the relationship between the number of 45S rDNA sites and the number of Ag-NOR signals (Figure 3D). As expected, there was a positive correlation between both variables (Spearman’s test, *p* < 0.05 (Table S4)). We also analyzed the number of Ag-NORs in groups differing in number of loci (Figure 4). 

In a group with two rDNA sites per diploid (corresponding to median number), the number of Ag-NOR signals roughly corresponds to the number of loci. However, as the number of loci increases, the relative proportion of Ag-NOR signals decreases. These results are correlated with those obtained at the genus level and involving smaller data sets. For example, in grasshoppers with a relatively high number of rDNA loci and genes [19], the number of inactive loci (Ag-NOR negative) ranged 12.7–22% [83]. Thus, despite a positive correlation between the number of 45S loci and number of Ag-NOR signals, the relationship is not a linear one. Karyotypes with a low and intermediate number of loci frequently show nearly all rDNA loci as active, whereas only less than half of them are usually active in karyotypes with a high number of loci. 

## 4. Conclusions

The updated version of the animal rDNA loci database (http://www.animalrdnadatabase.com/) presents a source of information about rDNA loci in a wide range of animal genera. Its new options allow the retrieval of information about the expression activity of rDNA loci and their position on different chromosome types. The animal rDNA loci database is a part of a long-term project also involving a mirror plant rDNA loci database (http://www.plantrdnadatabase.com/). We hope these regularly updated resources will be useful for evolutionary and structural analyses. Considering that the database’s coverage of information about the number and position of rDNA loci in chromosomes is still fragmentary (less than 0.2% species), to ensure that such comparative analyses are robust, there is clearly an ongoing need for high-quality cytogenetic studies, particularly in less represented groups, to make the data more comprehensive of animal diversity as a whole.

## Figures and Tables

**Table 1:** Amount of cytogenetic data for 5S and 45S rDNA loci in the database and their relationship to each group’s diversity.

Group	Group Diversity ^1^	rDNA locus	Number of Karyotypes	Number of Species	Species Repr. [%] ^2^
Invertebrates	~1.5 × 10^6^	5S	174	174	0.01
		45S	1173	1149	0.08
Vertebrates	~7.3 × 10^4^	5S	897	880	1.21
		45S	1557	1526	2.09
Total	~1.6 × 10^6^	5S	1071	1054	0.07
		45S	2730	2675	0.17

^1^ Number of species in the group. Source: IUCN [25]. ^2^ Percentages are counted as number of species/group diversity.

**Table 2:** Frequency of 5S and 45S rDNA localization on sex chromosomes.

Group	Karyotypes			Karyotypes with 45S				Karyotypes with 5S			
	Total	with Sex chr.		Total	45S on Sex chr.			Total	5S on Sex chr.		
	Nt	N_sex_	[%] ^1^	N_45_	N_45sex_	[%] ^2^	[%] ^3^	N_5_	N_5sex_	[%] ^4^	[%] ^5^
Amphibians	145	17	11.7	133	4	3	23.5	30	1	3.3	5.9
Arthropods	1068	733	68.6	1057	153	14.5	20.9	112	38	33.9	5.2
Birds	83	83	100	83	0	0	0	8	1	12.5	1.2
Fish	953	104	10.9	892	29	3.3	27.9	800	14	1.8	13.5
Mammals	275	275	100	263	14	5.3	5.1	44	5	11.4	1.8
Mollusks	80	2	2.5	80	0	0	0	50	0	0	0
Reptiles	175	70	40	175	7	4	10	13	0	0	0

^1^ Frequency of karyotypes with sex chromosomes calculated as: 100 × N_sex_/Nt. ^2^ Frequency of karyotypes with 45S rDNA on sex chromosomes calculated as: 100 × N_45sex_/N_45_. ^3^ Proportion of karyotypes with sex chromosomes bearing 45S rDNA loci on them: 100 × N_45sex_/N_sex_. ^4^ Frequency of karyotypes with 5S rDNA on sex chromosomes calculated as: 100 × N_5sex_/N_5_. ^5^ Proportion of karyotypes with sex chromosomes bearing 5S rDNA loci on them: 100 × N_5sex_/N_sex_.

**Table 3:** Frequency of 5S and 45S rDNA localization on B chromosomes.

Group	Total	Species with B chromosomes			Species with 5S or 45S rDNA on B chromosomes		
	N	N	[%]	Range	N	[%]	Fraction [%] ^1^
Amphibians	145	4	2.8	0–4	1	0.7	25.0
Arthropods	1068	56	5.2	0–14	7	0.7	15.5
Fish	953	34	3.6	0–10	13	1.4	38.2
Flatworm	14	1	7.1	0–4	0	0.0	0
Mammals	275	9	3.3	0–30	2	0.7	22.2
Mollusks	80	1	1.3	0–3	0	0.0	0
Reptiles	175	2	1.1	0–2	0	0.0	0

^1^ Percentages are calculated as the number of species with rDNA on B chromosomes (column 6) divided by the number of species harboring B chromosomes (column 3).

**Table 4:** Frequency of 45S rDNA localization in microchromosomes.

Group	Total	Species with Microchromosomes			rDNA loci in Microchromosomes		
		N	[%]	Range (Median)	N	[%]	Fraction [%]
Amphibians	145	6	4.1	6–50 (36)	4	2.8	66.7
Birds	83	83	100.0	6–72 (62)	71	85.5	85.5
Lamprey ^1^	3	3	100.0	164–168 (168)	3	3	100.0
Fish	953	6	0.6	3–44 (20)	0	0	0
Reptiles	175	125	71.4	2–50 (22)	35	20.2	28.0

^1^ Lampreys represent the most ancient lineage of modern vertebrates. Their chromosomes are typically numerous (2n = 144–188), and small (0.5–5.0 µm) chromosomes are often regarded as microchromosomes [35]. Chromosome morphologies could be distinguished for some of them.

## Data Availability

All data regarding results are available in the supplementary information and online at http://www.animalrdnadatabase.com/.

## Supplementary Materials

The following files are available online at https://www.mdpi.com/article/10.3390/ijms222111403/s1. Table S1: Statistical evaluation of the number of 5S and 45S rDNA loci in karyotypes. Table S2: Statistical comparison of the 5S and 45S average locus numbers using Mann–Whitney and ANOVA one-way tests. Page 1—45S rDNA analyses. Page 2—5S rDNA analyses. Table S3: Statistical evaluation of rDNA loci variability between the groups (Levene’s test, [24]). Table S4: Correlations between the rDNA locus numbers and other variables including number of chromosomes and number of Ag-NOR signals. Table S5: Comparison of the frequency of 45S rDNA loci on sex chromosomes between the main groups of animals (chi-square test). Table S5: Statistical evaluation of the 45S rDNA locus numbers and number of Ag-NOR signals between the groups. Figure S1. Venn diagram showing the proportion of 45S and 5S loci on sex chromosomes in karyotypes with known positions for both loci and where either 5S or 45S occur on a sex chromosome. N is the number of species.
