# Supplementary material for: Analyses of the Updated “Animal rDNA Loci Database” with an Emphasis on Its New Features
Source: Int J Mol Sci. 2021 Oct 22;22(21):11403. doi: 10.3390/ijms222111403 (PMC8584138; doi:10.3390/ijms222111403)
Supplement: Supplementary file 1 [file ijms-22-11403-s001.zip › Supplementary Tables_S1.pdf]

Table S1. Statistical evaluation of number of 5S and 45S rDNA loci in karyotypes

**Taxonomy**		**5S rDNA**						**45S rDNA**					
		**N**	**minimum**	**maximum**	**average**	**s.d.**	**median**	**N**	**minimum**	**maximum**	**average**	**s.d.**	**median**
vertebrate	fish	800	1	54	4.24	4.67	2.00	892	1	54	3.45	3.91	2.00
	mammals	44	2	18	2.77	2.50	2.00	263	1	42	5.51	6.31	3.00
	amphibians	30	2	28	4.33	3.59	2.00	133	1	12	2.43	1.33	2.00
	reptiles	13	2	74	18.18	25.10	2.00	175	1	7	2.14	0.50	2.00
	lampreys							3	4	18	9.33	6.18	6.00
	cartilaginous fish	2	4	4	4.00	0.00	4.00	8	2	16	5.00	4.37	3.75
	birds	8	2	2	2.00	0.00	2.00	83	2	13	2.78	1.85	2.00
invertebrate	arthropods	112	1	40	6.46	7.79	2.25	1057	1	30	3.14	2.65	2.00
	mollusks	50	2	10	3.58	2.31	2.00	80	2	9	3.13	1.84	2.00
	annelid							10	2	6	2.55	0.91	2.00
	flatworm	6	4	5	3.50	1.12	4.00	14	2	8	3.39	2.02	2.00
	thorny-head-worms							2	4	4	4.00	0.00	4.00
	cnidaria	3	2	2	2.00	0.00	2.00	8	2	5	2.63	1.11	2.00

Groups used for further statistical analyses (Tables S2, S3 and S4) are highlighted
s.d. - standard deviation
