# Supplementary material for: Analyses of the Updated “Animal rDNA Loci Database” with an Emphasis on Its New Features
Source: Int J Mol Sci. 2021 Oct 22;22(21):11403. doi: 10.3390/ijms222111403 (PMC8584138; doi:10.3390/ijms222111403)
Supplement: Supplementary file 1 [file ijms-22-11403-s001.zip › Supplementary Tables_S2.pdf]

Table S2. Statistical comparison of the 5S and 45S average locus numbers using Mann-Whitney and ANOVA 1-way tests

Mann-Whitney U test

45S rDNA

	P value						
	Amphibians	Arthropods	Birds	Fish	Mammals	Mollusks	Reptiles
Amphibians	-	3.87E-03	0.21	4.76E-05	9.72E-13	1.48E-04	9.98E-02
Arthropods		-	0.35	0.02	1.33E-15	0.08	4.07E-07
Birds			-	0.06	7.91E-07	0.03	1.85E-03
Fish				-	2.15E-11	0.37	5.79E-11
Mammals					-	1.96E-03	1.11E-16
Mollusks						-	9.48E-10
Reptiles							-

ANOVA 1-way test

45S rDNA

		P value						
		Amphibians	Arthropods	Birds	Fish	Mammals	Mollusks	Reptiles
F	Amphibians	-	2.31E-03	0.11	2.90E-03	5.04E-08	1.78E-03	8.05E-03
	Arthropods	9.33	-	0.22	0.04	-6.66E-16	0.95	6.46E-07
	Birds	2.60	1.49	-	0.12	1.25E-04	0.24	2.83E-05
	Fish	8.91	4.30	2.39	-	2.09E-10	0.46	1.02E-05
	Mammals	30.89	87.09	15.05	41.12	-	9.51E-04	7.27E-12
	Mollusks	10.02	0.00	1.38	0.56	11.11	-	3.28E-10
	Reptiles	7.12	25.03	18.18	19.67	49.63	42.84	-

Mann-Whitney U test

5S rDNA

	P value			
	Arthropods	Fish	Mammals	Mollusks
Arthropods	-	0.07	5.27E-04	0.17
Fish		-	7.19E-04	0.77
Mammals			-	0.01
Mollusks				-

ANOVA 1-way test

5S rDNA

		P value			
F		Arthropods	Fish	Mammals	Mollusks
	Arthropods	-	2.20683E-05	2.59E-03	0.0116015
	Fish	18.19123	-	0.0384577	0.318481
	Mammals	9.381599	4.298139	-	0.111008
	Mollusks	6.519994	0.996346	2.5894	-

The P and F values are above and below the diagonal, respectively
The P values below 0.05 are in red
