# Supplementary material for: Analyses of the Updated “Animal rDNA Loci Database” with an Emphasis on Its New Features
Source: Int J Mol Sci. 2021 Oct 22;22(21):11403. doi: 10.3390/ijms222111403 (PMC8584138; doi:10.3390/ijms222111403)
Supplement: Supplementary file 1 [file ijms-22-11403-s001.zip › Supplementary Tables_S3.pdf]

Table S3. Statistical evaluation of rDNA loci variability between the groups (Levene's test).

45S rDNA

	Amphibians	Arthropods	Birds	Fish	Mammals	Mollusks	Reptiles
Amphibians	-	0.00190966	0.159373	0.0042522	7.97277E-09	0.003266	0.00241842
Arthropods	9.677662	-	0.159429	0.0798014	0	0.810312	1.5289E-07
Birds	1.994027	1.98223	-	0.120365	3.82073E-05	0.241583	0.000032885
Fish	8.209622	3.07215	2.416805	-	7.53E-11	0.451384	1.04723E-05
Mammals	34.773514	90.774173	17.409197	43.184004	-	0.00039	1.99618E-13
Mollusks	8.854008	0.0576379	1.381491	0.567632	12.835593	-	4.00545E-10
Reptiles	9.356518	27.874818	17.87318	19.610751	57.557363	42.38428	-

The P and F values are above and below the diagonal, respectively
The P values below 0.01 are in red

5S rDNA

	Arthropods	Fish	Mammals	Mollusks
Arthropods	-	1.6215E-05	1.99E-03	0.0094283
Fish	18.790627	-	0.0378947	0.315575
Mammals	9.896159	4.323361	-	0.179036
Mollusks	6.905733	1.00839	1.83346	-

The P and F values are above and below the diagonal, respectively
The P values below 0.01 are in red
