# Supplementary material for: Analyses of the Updated “Animal rDNA Loci Database” with an Emphasis on Its New Features
Source: Int J Mol Sci. 2021 Oct 22;22(21):11403. doi: 10.3390/ijms222111403 (PMC8584138; doi:10.3390/ijms222111403)
Supplement: Supplementary file 1 [file ijms-22-11403-s001.zip › Supplementary tables_S4.pdf]

Table S4. Correlations between the rDNA locus numbers and other variables including number of chromosomes and number of Ag-NOR signals

Variable X	Variable Y		Spearman	
		N[1]	Rs	2-sided P-value
5S rDNA	chromosome number (2N)	1062	0.015796	0.606947866
45S rDNA	chromosome number (2N)	2709	0.123869	9.85932E-11
5S rDNA	45S rDNA	974	0.223007	1.92633E-12
Ag-NOR	45S rDNA	1193	0.760805	7.08E-226

[1] Number of pairwise comparisons. All distributions deviated from normality (Jarque-Bera Normality Test)
