# Supplementary material for: Analyses of the Updated “Animal rDNA Loci Database” with an Emphasis on Its New Features
Source: Int J Mol Sci. 2021 Oct 22;22(21):11403. doi: 10.3390/ijms222111403 (PMC8584138; doi:10.3390/ijms222111403)
Supplement: Supplementary file 1 [file ijms-22-11403-s001.zip › Supplementary Tables_S5.pdf]

**Table S5.** Comparison of the frequency of 45S rDNA loci on sex chromosomes between the main groups (chi square test).

| **Group** | **Species with sex chromosomes** | **45S rDNA on sex chromosomes** | **Percentage** |
|---|---|---|---|
| Arthropods | 733 | 153 | 20.9 |
| Fish | 104 | 29 | 27.9 |
| Mammals | 275 | 14 | 5.1 |

**The chi square statistics. The null hypothesis states that the number of loci between the groups are the same.**

Comaprison of Arthropods versus fish

The chi-square statistic is 2.1562. The $p$ -value is .141994. *Not* significant at $p < .05$.

The chi-square statistic with Yates correction is 1.8337. The $p$ -value is .175686. *Not* significant at $p < .05$.

Comparison of Arthropods versus mammals

The chi-square statistic is 29.7448. The $p$ -value is < 0.00001. Significant at $p < .05$.

The chi-square statistic with Yates correction is 28.7524. The $p$ -value is < 0.00001. Significant at $p < .05$.

Comparison of fish versus mammals

The chi-square statistic is 206.9852. The $p$ -value is < 0.00001. Significant at $p < .05$.

The chi-square statistic with Yates correction is 204.4435. The $p$ -value is < 0.00001. Significant at $p < .05$.
