# Supplementary material for: Analyses of the Updated “Animal rDNA Loci Database” with an Emphasis on Its New Features
Source: Int J Mol Sci. 2021 Oct 22;22(21):11403. doi: 10.3390/ijms222111403 (PMC8584138; doi:10.3390/ijms222111403)
Supplement: Supplementary file 1 [file ijms-22-11403-s001.zip › Figure S1.pdf]

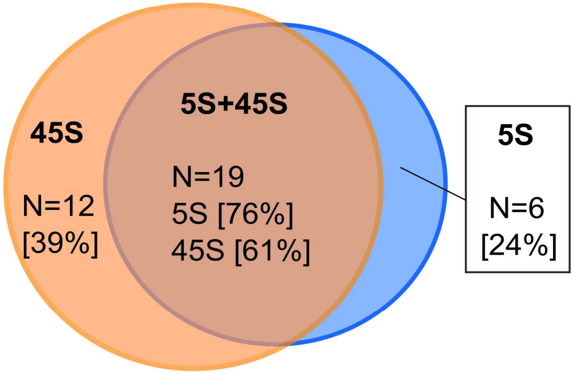

Figure S1. Venn diagram showing the proportion of 45S and 5S loci on sex chromosomes) in karyotypes with known position for both loci and where either 5S or 45S occur on a sex chromosome. N is the number of species.
